# In Vivo and In Vitro Suppression of Hepatocellular Carcinoma by EF24, a Curcumin Analog

**DOI:** 10.1371/journal.pone.0048075

**Published:** 2012-10-31

**Authors:** Haitao Liu, Yingjian Liang, Luoluo Wang, Lantian Tian, Ruipeng Song, Tianwen Han, Shangha Pan, Lianxin Liu

**Affiliations:** 1 Department of Hepatic Surgery, The First Affiliated Hospital of Harbin Medical University, Key Laboratory of Hepatosplenic Surgery, Ministry of Education, Harbin, Heilongjiang Province, P.R.China; 2 Research Centre of The First Affiliated Hospital of Harbin Medical University, Harbin, Heilongjiang Province, P.R.China; Vanderbilt University Medical Center, United States of America

## Abstract

The synthetic compound 3,5-bis(2-flurobenzylidene)piperidin-4-one (EF24) is a potent analog of curcumin that exhibits enhanced biological activity and bioavailability without increasing toxicity. EF24 exerts antitumor activity by arresting the cell cycle and inducing apoptosis, suppressing many types of cancer cells in vitro. The antiproliferative and antiangiogenic properties of EF24 provide theoretical support for its development and application to liver cancers. We investigated the in vitro and in vivo activities of EF24 on liver cancer to better understand its therapeutic effects and mechanisms. EF24 induced significant apoptosis and G2/M-phase cell cycle arrest in mouse liver cancer cell lines, Hepa1-6 and H22. The expression levels of G2/M cell cycle regulating factors, cyclin B1 and Cdc2, were significantly decreased, pp53, p53, and p21 were significantly increased in EF24-treated cells. In addition, EF24 treatment significantly reduced Bcl-2 concomitant with an increase in Bax, enhanced the release of cytochrome *c* from the mitochondria into the cytosol, resulting in an upregulation of cleaved-caspase-3, which promoted poly (ADP-ribose) polymerase cleavage. EF24-treated cells also displayed decreases in phosphorylated Akt, phosphorylated extracellular signal-regulated kinase and vascular endothelial growth factor. Our in vitro protein expression data were confirmed in vivo using a subcutaneous hepatocellular carcinoma (HCC) tumor model. This mouse HCC model confirmed that total body weight was unchanged following EF24 treatment, although tumor weight was significantly decreased. Using an orthotopic HCC model, EF24 significantly reduced the liver/body weight ratio and relative tumor areas compared to the control group. In situ detection of apoptotic cells and quantification of Ki-67, a biomarker of cell proliferation, all indicated significant tumor suppression with EF24 treatment. These results suggest that EF24 exhibits anti-tumor activity on liver cancer cells via mitochondria-dependent apoptosis and inducing cell cycle arrest coupled with antiangiogenesis. The demonstrated activities of EF24 support its further evaluation as a treatment for human liver cancers.

## Introduction

Hepatocellular carcinoma (HCC) is the most common form of primary hepatic carcinoma, the fifth most common cancer, and the third leading cause of cancer-related deaths in the world [1], [2], [3]. HCC poses a sociomedical problem particularly in Asia and sub-Saharan Africa, where the number of deaths nearly equal the number of cases diagnosed annually (about 600,000), and the 5-year survival rate is below 9% [4], [5], [6], [7]. Several treatment options exist for HCC, including resection, liver transplantation, percutaneous ablation. However, the cure rate for patients who undergo resection is relatively low, and among patients who are ineligible for surgical or percutaneous procedures, only chemoembolization improves survival. Moreover, HCC is widely regarded as a chemotherapy-resistant disease [8], [9], [10], [11], [12]. These drawbacks necessitate the continued search for novel HCC therapies.

A diverse array of phytochemicals, including some obtained from fruits, vegetables, nuts, and spices, have demonstrated the capacity to selectively kill tumor cells and suppress carcinogenesis in preclinical animal models [13], [14], [15], [16], [17]. In several high-risk populations (e.g., patients with cardiovascular disease or cancer), phytochemicals have been shown to significantly prevent or delay cancer development [18], [19], [20]. Curcumin, a polyphenol extracted from rhizomes of Curcuma longa L., is a well-known chemopreventative agent that exhibits potent anticarcinogenic activity in a wide variety of tumor cells. Curcumin shows significant therapeutic potential for liver cancers because it suppresses cancer cell proliferation, induces cell cycle arrest and apoptosis via the caspase cascade, and inhibits hypoxia-inducible factor-1 (HIF-1) by degrading the aryl hydrocarbon receptor nuclear translocator. Curcumin also exerts anticarcinogenic effects by decreasing the expression of cyclooxygenase-2 (COX-2) and vascular endothelial growth factor (VEGF) [21], [22], [23], [24], [25], [26]. Unfortunately, curcumin’s therapeutic benefit is limited by very low absorptive capacity upon transdermal or oral application [27]. Ames et al. developed a series of novel synthetic curcumin analogs with higher potencies and improved water solubilities [28]. One of these compounds, EF24, exhibited approximately 10- and 20-fold enhanced cytotoxic activity against various cancer cell lines relative to curcumin and cisplatin, respectively [29]. EF24 induces G2/M phase cell cycle arrest and apoptosis by increasing phosphatase and tensin homolog (PTEN) expression in the human ovarian carcinoma cell line, A2780. Moreover, EF24 inhibits HIF transcriptional activity in MDA-MB231 breast cancer cells and in PC3 prostate cancer cells. Lung cancer cell viability is decreased by EF24 via increased phosphorylation of extracellular regulated kinases (ERK)1/2, c-Jun N-terminal kinases (JNK), and p38 mitogen-activated protein kinases (MAPK). EF24 also inhibits the proliferation of HCT-116 and HT-29 colon cancer cells as well as AGS gastric adenocarcinoma cells [30], [31], [32], [33].

Recent studies have suggested that EF24 may inhibit the proliferation of liver cancer cells by interfering with the nuclear factor kappa B (NF-kB) pathway. EF24 has demonstrated superior pharmacokinetic and activity profiles in animal models and is well-tolerated [29], [34]. In addition, the compound inhibits VEGF-induced angiogenesis in rabbit and mouse models and significantly reduces tumor sizes in athymic nude mice xenografted with human breast cancer tumors [35].

Considering EF24’s inhibition of ERK 1/2, upregulation of activated caspase-3, and alteration of Bax/Bcl-2 and Bax/Bcl-xL ratios [33], [36], we examined the therapeutic potential and molecular mechanisms of EF24 on liver cancer cells. EF24 potently induced apoptosis via mitochondrial pathway and arrested G2/M-phase cell cycle progression in mouse liver cancer cell lines, Hepa1-6 and H22. The expression levels of G2/M cell cycle regulating factors, pp53, p53, and p21 were significantly increased, cyclin B1 and Cdc2, were decreased with EF24 treatment, as were pAkt, pERK and VEGF. In vitro angiogenesis assay and in vivo measurements of liver weight, body weight, and tumor volume suggest that EF24 suppresses tumor growth and induces apoptosis.

## Results

### EF24 Inhibits Cell Proliferation and Reduces Cell Viability

**Figure 1:**
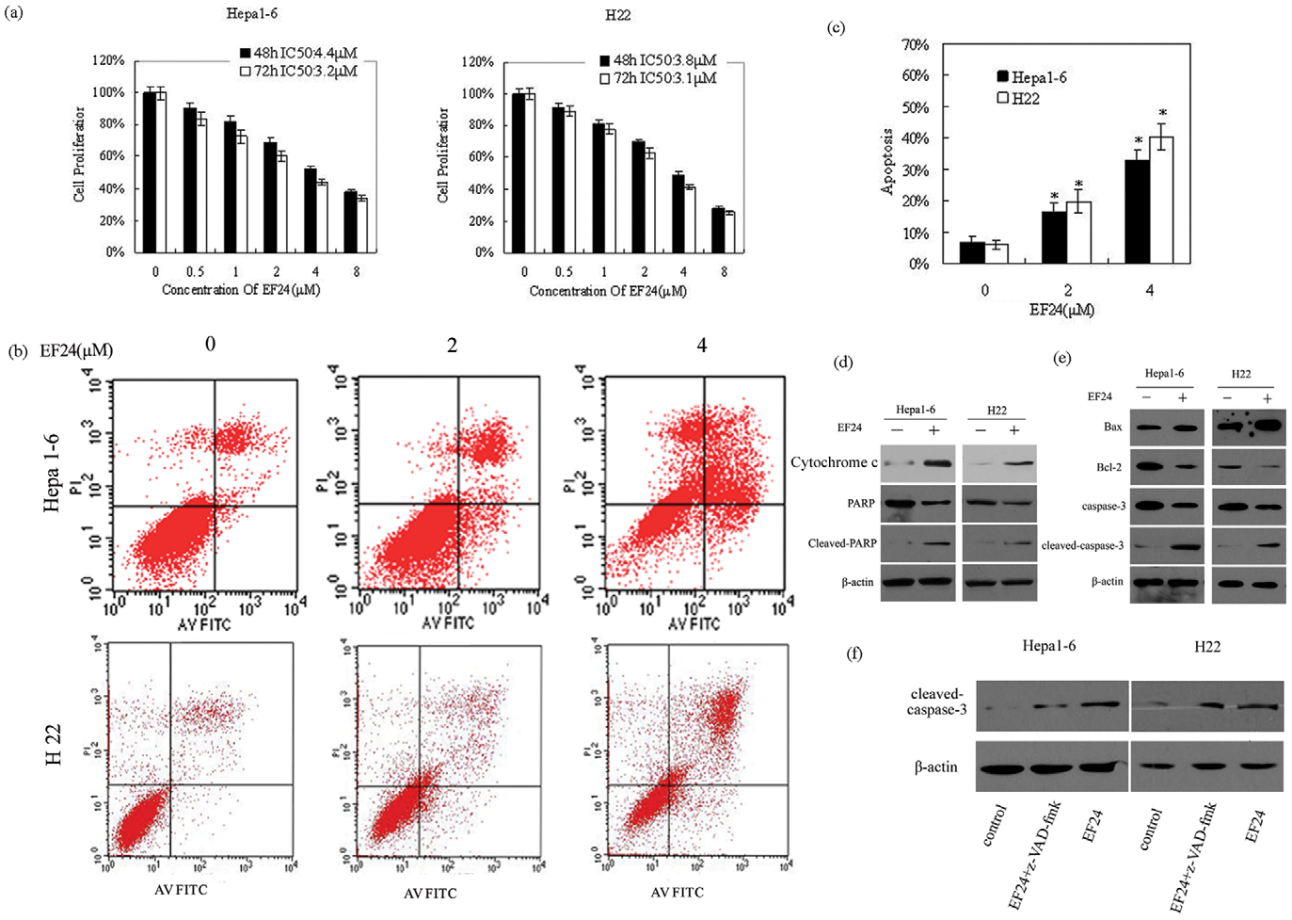
EF24 inhibits cell proliferation and reduces cell viability. (**a**). Hepa1-6 and H22 cells were treated with the indicated concentrations of EF24 for 48 h and 72 h. Cell growth was determined by cell counting kit-8 assay. (**b**). Hepa1-6 cells and H22 cells incubated with 2 µM and 4 µM of EF24 for 48 h were analyzed for apoptosis. (**c**). Percentage of apoptotic cells as determined by flow cytometry of three independent experiments, * P<0.01 compared with the untreated (DMSO) cells. (**d**). and (**e**). Lysates from Hepa1-6 and H22 cells incubated with EF24 (4 µM) were analyzed by Western blotting for apoptosis-related proteins. (**f**). Hepa1-6 and H22 cells treated with EF24 and EF24 in combination with pan-caspase inhibitor z-VAD-fmk, Cell lysates were used for immunoblot assay for cleaved caspase-3.

Using the CCK-8 assay, we evaluated the effect of EF24 on the proliferation of Hepa1-6 and H22 cells. At a dose of 4 µM, EF24 exposure for 48 h strongly inhibited cell proliferation, producing IC50 values of 4.4 µM for Hepa1-6 and 3.8 µM for H22 cells (Fig. 1a). To evaluate whether EF24 inhibited cell viability by inducing apoptosis, we performed an annexin V/propidium iodide assay. Within 48 h of EF24 treatment (4 µM), 40.4% of H22 cells and 31.8% of Hepa1-6 cells underwent apoptosis (Fig. 1b and c).

Liver cancer cells incubated with EF24 (4 µM) for 48 h were assessed by Western blotting using antibodies that recognize the intact (116 kDa) and cleaved (89 kDa) forms of PARP as well as other apoptosis-related proteins. The levels of cytochrome *c*, cleaved-PARP, Bax, and activated caspase-3 increased, whereas PARP and Bcl-2 were downregulated compared with non-EF24-treated controls (Fig. 1d and e). Treatment of Hepa1-6 and H22 cells with the pan-caspase inhibitor, z-VAD-fmk, indicated that the apoptosis induced by EF24 was at least partly caspase-dependent (Fig. 1f).

### EF24 Induces Cell Cycle Arrest in Mouse Liver Cancer Cells

**Figure 2:**
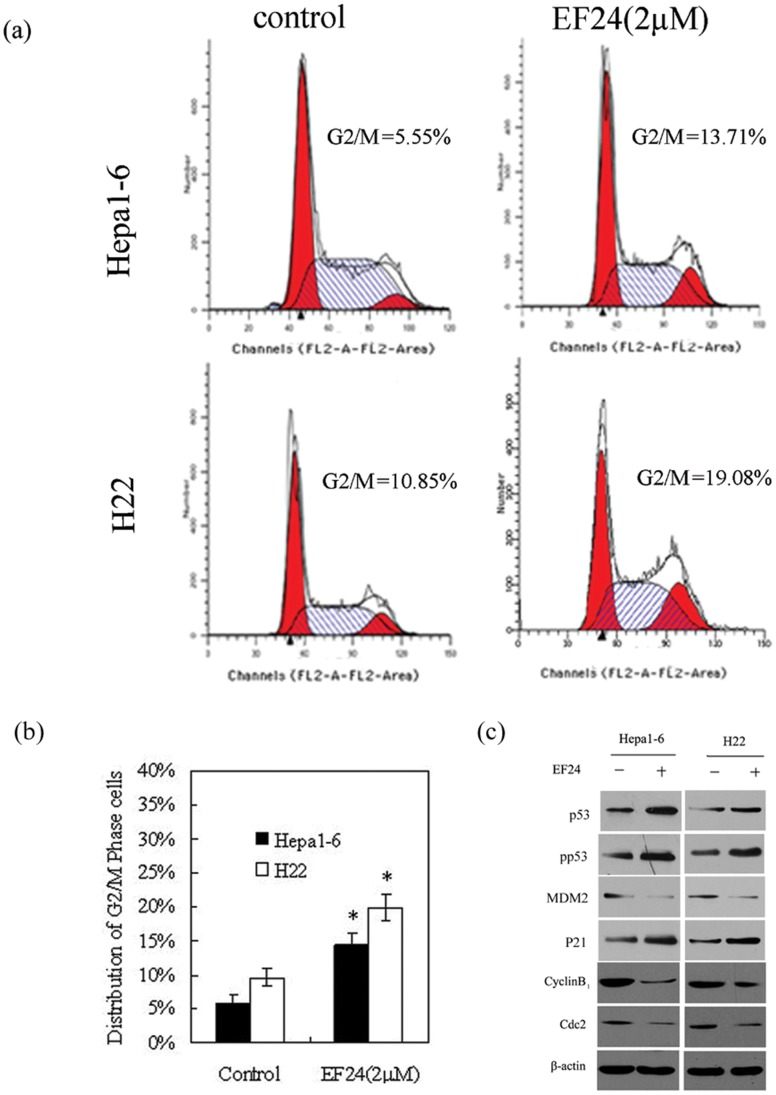
EF24 induces cell cycle arrest in mouse liver cancer. Cells were treated with either DMSO (control) or EF24 (4 µΜ) for 48 h were collected, flow cytometric analysis was performed for cell cycle distribution. (**a**). Representative flow cytometry graph for each treated or untreated groups. (**b**). Distribution of G2/M phase cells. Data represent mean ± SD of three independent experiments, *p<0.05 as compared with control group. (**c**). Immunoblot images of cell cycle regulatory molecules CycB1, Cdc2, p53, pp53, p21, MDM2 from treated (EF24, 4 µM, is denoted by “+”) and untreated (is denoted by “−”) cells.

Cell cycle analysis was performed to determine the stage at which EF24 arrests liver cancer cells. EF24-treated (2 µM) Hepa1-6 and H22 cells were fixed, and cell cycle distributions were determined by flow cytometry. EF24 treatment for 48 h arrested cells at the G2/M stage (Fig. 2a and b). Western blotting of G2/M cell cycle regulatory molecules demonstrated that cyclin B1 and Cdc2 were significantly reduced, pp53, p53, and p21 were significantly increased with EF24 treatment, In addition, the level of MDM2, one of the negative regulators of p53, was also decreased after EF24 treatment (Fig. 2c).

### EF24 Inhibits Angiogenesis and Tumor Cell Survival Signaling in Liver Cancer

**Figure 3:**
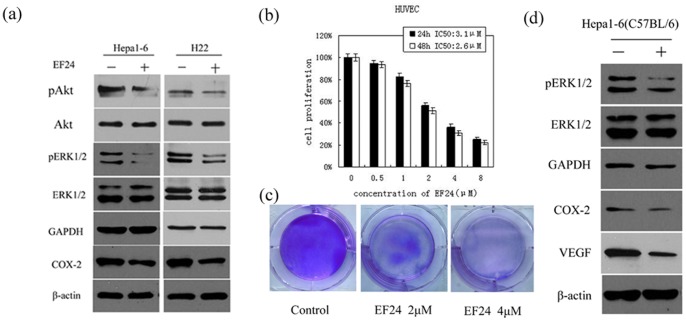
EF24 inhibits angiogenesis and tumor cell survival signaling in liver cancer. (**a**). Lysates from EF24-treated (4 µM,) cells (Hepa1-6 and H22) used for western blot analysis on the expressions of ERK, p-ERK, Akt, p-Akt and COX-2. (**b**). HUVEC cells was treated with the indicated concentrations of EF24 for 24 h and 48 h. Cell growth was determined by cell counting kit-8 assay. (**c**). The proliferation of cells was also measured by crystal violet assay. (**d**). Tumor tissue lysates was used for western blotting to detect the levels of p-ERK, COX-2 and VEGF.

Western blot analyses of Hepa1-6 and H22 cell lysates indicated a significant decrease in Akt and ERK phosphorylation status following EF24 treatment (4 µM), as was COX-2, which is involved in cell proliferation. In contrast, EF24 had no impact on the level of total Akt, ERK (Fig. 3a), p38, or JNK (data not shown). Human umbilical vein endothelial cells (HUVECs) were treated with different concentrations of EF24 for 24 or 48 h, before the cell proliferation was assessed by CCK-8 assay. As shown in Fig. 3b, At a dose of 2 µM, EF24 exposure for 48 h inhibited cell proliferation effectively, producing IC50 value of 3.1 µM and 2.6 µM after treatment with EF24 for 24 h and 48 h, respectively. EF24 inhibited the proliferation of HUVECs in a dose-dependent manner for 24 or 48 h. These results were confirmed by crystal violet assay (Fig. 3c). These studies suggest that EF24 may exhibit its antiangiogenic activity through inhibit the proliferation of specific growth-related signals of vascular endothelial cell.In vivo, EF24 significantly inhibited ERK phosphorylation (Fig. 3d). All these findings suggesting that EF24 maybe a potent inhibitor of tumor cell survival via PI3K/AKT, ERK-MAPK pathway inhibition. As expected from in vitro results, VEGF and COX-2 expression levels were significantly reduced in EF24-treated subcutaneous HCC tumor models (Fig. 3d).

### EF24 Inhibits Tumor Growth and Induces Apoptosis in a Subcutaneous HCC Tumor Model and in an Orthotopic HCC Model

**Figure 4:**
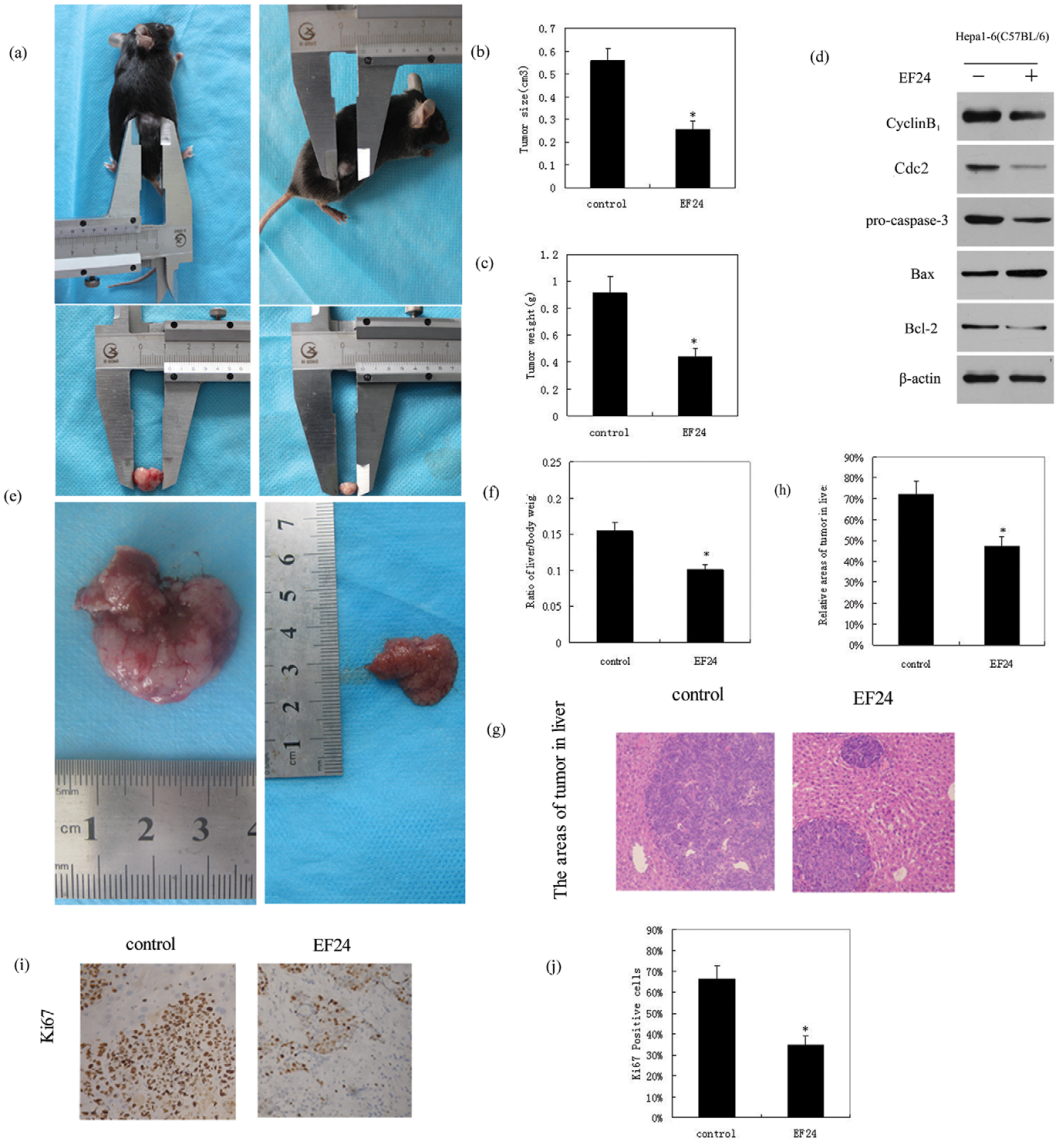
EF24 inhibits liver cancer growth and induces apoptosis both in subcutaneous HCC tumor model and orthotopic HCC models. (**a**). EF24 inhibits mice subcutaneous tumor growth in vivo. (**b**). Tumor size was measured after treated for three weeks. (**c**). EF24-treated animals resulted in a significantly lower tumor weight when compared with controls. (**d**). Tumor tissue removed from the animals were used for western blot analysis on the expressions of caspase-3, Cyclin B1, Cdc2, Bcl-2 and Bax. (**e**). Liver removed from the orthotopic HCC models, treated or untreated with EF24 for three weeks. (**f**). EF24 treatment groups significantly reduced the liver/body weight ratio compared to the control. (**g**). After treatmeant for three weeks, tumor tissue sections were stained with haematoxylin and eosion (HE). (**h**). Relative areas occupied by tumors in livers were calculated. (**i**). Tumor sections were stained with an anti-Ki-67 Ab to detect proliferating cells. (**j**). Cells expressing Ki-67 were counted to calculate the proliferation index, Assay was done in triplicate and p<0.01 is denoted by “*”.

EF24 treatment significantly inhibited tumor growth in the subcutaneous HCC model (Fig. 4a), but did not affect total body weight, relative to controls. EF24-treated animals resulted in a significantly lower tumor size and weight compared with controls (Fig. 4b and c). Western blot analysis of subcutaneous tumors excised on day 22 after EF24 treatment indicated decreased protein expression levels of caspase-3, cyclin B1, Cdc2, and the Bcl-2/Bax ratio, relative to controls (Fig. 4d).

In the orthotopic HCC model, mice were terminated after 3 weeks of treatment, and livers were excised (Fig. 4e). EF24-treated animals displayed a significantly reduced liver/body weight ratio (0.155), compared with controls (0.101) P<0.01(Fig. 4f). EF24 also significantly reduced the relative areas of the tumors (47%) compared with controls (72%) (Fig. 4g and h). Ki-67-based quantification of cell proliferation in EF24-treated tumors removed from orthotopic HCC animals indicated the presence of more apoptotic cells and fewer Ki-67 positive cells (Fig. 4i and j). These results suggest that EF24 significantly inhibits HCC tumor growth and induces apoptosis in vivo.

## Discussion

HCC is a common cancer typically associated with poor prognoses, we set up to investigate novel therapies for this disease, and find that EF24, a curcumin analogue, possesses great potential as a promising anti-HCC therapeutic agent. EF24 induces G2/M-phase cell cycle arrest in several different types of cancer cells, including human breast, prostate, and cisplatin-resistant ovarian cancer cells [30], [34]. We detected G2/M-phase cell cycle arrest in two mouse liver cancer cells lines following treatment with EF24. The G2/M-related proteins, cyclin B1 and Cdc2, were decreased following treatment, while the pp53, p53, and p21, were increased. The cyclinB1–Cdc2 complex is a key regulator of G2/M cell cycle checkpoints. Down-regulation of cyclin B1 and Cdc2 contributes critically to the G2/M-phase cell cycle arrest under conditions of DNA damage. Tumor suppressor protein of p53 controls the G2/M cell cycle checkpoints that mediate growth arrest [37], in our studies, we found that MDM2 was decreased after treatment, which is a negative regulator of p53. Its downregulation increases the transactivation of p53 to promote the induction of p21, which causes cell cycle arrest in the G2/M phase by binding of the cyclin-cdk complex [38], [39], [40].

EF24 can induce cell apoptosis via the mitochondrial cell death pathway. EF24-treated liver cancer cells expressed significantly lower levels of Bcl-2 while concomitantly upregulating Bax expression, compared with control cells. In addition, the release of cytochrome *c* from the mitochondria was found to be increased in the treatment group, which release was known to be facilitated by Bax and blocked by Bcl-2 [41]. This dynamic upregulated cleaved-caspase-3, promoting PARP cleavage, which is one of the hallmarks of apoptosis [42]. Notably, EF24-induced liver cancer cell death was not fully blocked by the general caspase inhibitor, z-VAD-fmk, suggesting that additional mechanisms contribute to EF24’s activity.

MAPKs including ERK, p38, and JNK, are activated by a wide array of extracellular signals that elicit phosphorylation cascades, transduce mitogenic signals to the nucleus, and modulate the activity of transcription factors [43], [44], [45], [46]. The isoforms p42mapk (ERK-2) and p44mapk (ERK-1) are directly activated by phosphorylation on specific tyrosine and threonine residues by the dual-specificity ERK kinase. Because curcumin can inhibit liver cancer cell growth by decreasing ERK activation [33], [47], we investigated whether the effect of EF24 on liver cancer cells was associated with ERK inhibition as well. Western blot analyses of Hepa1-6 and H22 cells lysates indicated significantly decreased ERK phosphorylation following EF24 treatment. In contrast, p38 and JNK expression were unaffected by EF24 treatment, suggesting that pathways other than ERK–MAPK may also be affected by EF24 in liver cancer cells. Our next phase of research is to examine activated ERK, which relays mitogenic signals to the nucleus and modulates the activity of transcription factors. In addition, we found the expression level of phospho-Akt was decreased, without any changes in the total Akt protein level following EF24 treatment. The downregulation of pAkt resulting in increased apoptosis and decreased cell survival as several reports have shown that some anticancer agents induced apoptosis partly by blocking the activation of Akt [48], [49].

Tumor angiogenesis, a prerequisite for tumor growth, involves several types of growth factors. Of these, VEGF is the most powerful, it is a potent inducer of capillary growth into a tumor, and without angiogenesis, tumor growth typically is limited to 1–2 mm [33]. A study involving immunohistochemistry analysis of 24 HCC cases reported that COX-2 and VEGF expression levels were positively correlated [50]. COX-2 inhibition induces apoptosis signaling via death receptors in HCC [51]. In vivo, We observed a significant decrease in both COX-2 and VEGF expression. In vitro, we also found that treatment of HUVECs with EF24 result in a significant inhibition of the cell proliferation, suggesting that EF24 may exhibit the antiangiogenic activity by inhibition of vascular endothelial cell proliferation, or this pathway is involved in EF24-mediated HCC suppression.

To determine whether EF24 was superior to curcumin in vivo, we designed and executed a subcutaneous HCC tumor model using C57BL/6 mice. After 3 weeks of treatment, we measured tumor volume and tumor weight, and observed that EF24 evoked a significant suppression of tumor growth compared with controls, and our novel orthotopic HCC model supports our subcutaneous HCC tumor model. However, EF24 treatment did not affect total body weight. Excised tumors were subsequently examined for consistency with our in vitro protein expression results. Indeed, we detected decreased in vivo expression of p-ERK, pro-caspase-3, VEGF, COX-2, cyclin B1, Cdc2, and Bcl-2/Bax in the EF24-treated group. These data support EF24 as a potential therapeutic for liver cancer cells and indicate that EF24 functions via multiple molecular targets to suppress cancer cell proliferation and antiangiogenesis and induce cell cycle arrest and apoptosis.

Our results support the further development of EF24 as a liver cancer treatment. EF24 promotes apoptosis through a cascade reaction and induces cell cycle arrest coupled with anti-angiogenesis. Our study was the first to report a reduction in p-ERK following EF24 treatment of liver cancer cells, and our orthotopic HCC model evaluated for the first time the therapeutic effect of EF24 on liver cancer cells. EF24 significantly inhibits liver cancer cells in vivo and in vitro, indicating that EF24 is an anti-tumor drug worthy of further investigation and clinical evaluation.

## Materials and Methods

### Animals, Cell Lines, Reagents, and Antibodies

Male C57BL/6 mice (6–8 weeks old) were purchased from the Experimental Animal Research Center at The First Affiliated Hospital of Harbin Medical University, China. All operative procedures were approved by the Institutional Ethics Committee at Harbin Medical University. All experiments were performed in accordance with the guidelines of the Committee on the Use of Live Animals in Teaching and Research of the Harbin Medical University, Harbin, China. Cell lines, H22 (China Center for Type Culture Collection, Wuhan, China), HUVEC cells (American Type Culture Collection, Rockville, MD, USA) were cultured in RPMI 1640 and Hepa1-6 (from a C57BL/6 mouse hepatoma [H-2b]; American Type Culture Collection, Rockville, MD, USA) was cultured in Dulbecco’s modified Eagle’s medium (DMEM), all supplemented with 10% fetal calf serum and 1% penicillin-streptomycin solution at 37°C and 5% CO2. RPMI 1640 medium, DMEM, fetal calf serum, antibiotics, trypsin-EDTA, and phosphate-buffered saline (PBS) were purchased from Gibco BRL (Grand Island, NY, USA). EF24 was synthesized as reported by Adams et al. [29]. Primary antibodies against Bcl-2, Bax, caspase-3, cleaved-caspase-3, p53, pp53, pAkt, Akt, cytochrome c, MDM2, cyclin B1, GAPDH and secondary antibodies against mouse IgG-horse radish peroxidase (HRP) and rabbit IgG-HRP were obtained from Santa Cruz Biotechnology, Inc. (Santa Cruz, CA, USA). Antibodies against PARP, p38, JNK, cleaved- poly (ADP-ribose) polymerase (PARP), p44/42 MAPK (ERK 1/2), phospho-p44/42 MAPK (p-T202, p-Y204) (p-ERK), Cdc2, Ki67, and COX-2 were purchased from Cell Signaling Technology, Inc. (Trask Lane, Danvers, MA, USA). Antibody against VEGF was acquired from Abcam Inc. (Cambridge, MA, USA).

### Cell Viability Assay

Hepal-6 and H22 cells were seeded into 96-well plates at densities of 3×10^3^ cells/well and were cultured for 48 h or 72 h in DMEM (HUVECs were cultured for 24 h or 48 h in RPMI1640) supplemented with 10% FBS and containing EF24 at various concentrations (0, 0.5, 1.0, 2.0, 4.0, or 8.0 µM ).Cells viability was assessed using a Cell Counting Kit-8 (CCK-8; Dojindo Molecular Technologies, Tokyo, Japan). The cell viability index was calculated as a percentage of the absorbance in treated wells relative to the absorbance in untreated (control) wells. Crystal violet assay method: HUVECs (1×10^5^) were seeded into 6-well plates and cultured overnight. Then cells was cultured in the medium which containing EF24 (0, 2.0, or 14 µM) for 24 h or 48 h, then, cells were washed with PBS twice, the remaining cells were stained for 1 h with crystal violet solution (0.5% crystal violet, 20% methanol). The 6-well plates were washed with PBS three times and left to dry at 37°, pictures were taken. Experiments were conducted at least in triplicate.

### Apoptosis Assay

Hepal-6 and H22 cells (3×10^5^ cells/well) were cultured with EF24 (2 µM or 4 µM) for 48 h, 1×10^6^ cells were collected and washed twice with ice-cold PBS, suspended in binding buffer (100 µL) (BD Biosciences, San Jose, CA, USA), treated with Annexin V and Propidium iodide (PI) (BD Biosciences), and incubated in the dark for 15 min, anther 300 µL binding buffer were added, then flow cytometry analysis was performed within 1 h to measure the apoptosis rate (%).

### Cell Cycle Analysis

To determine the effects of EF24 on the cell cycle, Hepal-6 or H22 cells were incubated with EF24 (2 µM) for 48 h or 72 h. Cells were trypsinized to collect both suspended and previously adhered cells. Samples were washed twice with PBS, and the percentages of cells in G0/G1, S, and G2/M phases were determined using a cell cycle detection kit (BD Biosciences) in a Beckman Coulter EPICS ALTRA II cytometer (Beckman Coulter, CA, USA).

### Western Blotting

EF24-treated and -untreated Hepal-6 and H22 cells or tumor tissues removed from subcutaneous HCC tumor model were homogenized in protein lysate buffer, and debris was removed by centrifugation at 12,000 rpm for 10 min at 4°C. Proteins (50 µg/lane) were separated by 12% sodium dodecyl sulfate-polyacrylamide gel electrophoresis. Proteins were electrotransferred onto polyvinylidene fluoride membranes (Millipore), and membranes were washed with Tris-buffered saline (10 mM Tris, 150 mM NaCl) containing 0.05% Tween-20 and blocked with 3% bovine serum albumin. Membranes then were incubated with primary antibody (diluted 1∶1000) overnight, washed in TBST for 30 min, exposed to HRP-conjugated secondary antibody (diluted 1∶2000), and washed again in TBST for 30 min. Final detection was performed using enhanced chemiluminescence (Amersham Pharmacia Biotech, Buckinghamshire, UK).

### Subcutaneous HCC Tumor Model

Approximately 2×10^6^ Hepa1-6 cells in 200 µL PBS were injected subcutaneously into each animal’s back (n = 20). EF24 was dissolved in dimethyl sulfoxide and diluted in sodium chloride. Ten days post-injection, EF24-treated mice (n = 10) were injected intraperitoneally (i.p.) with 200 µL EF24 at a dose of 20 mg/kg/d for 21 d. Control mice (n = 10) were injected i.p. with 200 µL PBS daily. On day 22, tumors were removed and weighed. Tumor volumes were estimated according to the formula π/6×a^2^×b, where a is the short axis, and b is the long axis [52].

### Orthotopic HCC Model

Hepa1-6 cells were harvested following brief treatment with 0.25% trypsin and 0.2% EDTA. Trypsinization was stopped by adding medium containing 10% FBS. Cells then were washed in serum-free medium and resuspended in PBS. Cells with more than 90% viability were used for the injections. Mice were anesthetized with pentobarbital sodium, and a small left abdominal flank incision was made. Hepa1-6 cells (2×10^6^ in 100 µL PBS) then were injected into the spleen parenchyma. To avoid intrasplenic tumor growth, the spleen was removed 10 min post-injection. The incision then was closed in two layers, using vicryl 5/0 (Warwick Medical Supplies Company Limited, Hangzhou, China) for the abdominal wall and vicryl 4/0 for the skin. One week post-operation, the EF24-treated group was injected i.p. with 200 µl EF24 at a dose of 20 mg/kg/d, and control animals were injected i.p. with 200 µl PBS daily. Mice were sacrificed at 22 d, and weights of whole bodies and dissected livers were recorded.

### Tumor-occupied Areas in Excised Livers

Excised liver tissues were embedded in paraffin, cut to 5-µm-thick sections at 5 different liver depths, and stained with hematoxylin and eosin. Samples were visualized under 100× magnification (4 random fields/section; n = 20 fields). The relative areas occupied by the tumors were calculated according to the following formula: sum of tumor areas/20 field areas ×100% [53].

### Immunohistochemistry

To quantify tumor cell proliferation, tumor tissues were embedded in paraffin and cut to 5-µm-thick sections, immunostained with anti-Ki-67 antibody as described previously [52]. Positively staining cells from 3 tumors per group were counted in 10 randomly selected fields under 400× high-power magnification. A proliferative index (%) was calculated according to the following formula: number of Ki-67-positive cells/total cell count × 100%.

### Statistical Analysis

Data are presented as means ± standard deviations (SD) of three independent experiments. Statistical significance was determined using Student’s t-test or Analysis of Variance (ANOVA). Statistical significance was assigned for P<0.05.

## References

[1] Nakashima T, Okuda K, Kojiro M, Jimi A, Yamaguchi R, et al (1983) Pathology of hepatocellular carcinoma in Japan. 232 Consecutive cases autopsied in ten years. Cancer 51: 863–877. 6295617 10.1002/1097-0142(19830301)51:5<863::aid-cncr2820510520>3.0.co;2-d

[2] Jeal A, Ward E, Hao Y, Thun M (2005) Trends in the leading causes of death in the United States, 1970–2002. Journal of the American Medical Association 294: 1255–1259. 16160134 10.1001/jama.294.10.1255

[3] Ferenci P, Fried M, Labrecque D, Bruix J, Sherman M, et al (2010) Hepatocellular carcinoma (hcc): a global perspective. J Clin Gastroenterol 44: 239–245. 20216082 10.1097/MCG.0b013e3181d46ef2

[4] Bosch FX, Ribes J, Díaz M, Cléries R (2004) Primary liver cancer: worldwide incidence and trends. Gastroenterology 127: S5–S16. 15508102 10.1053/j.gastro.2004.09.011

[5] Sherman M (2005) Hepatocellular cancinoma: epidemiology, risk factors, and screening. Semin Liver Dis 25: 143–154. 15918143 10.1055/s-2005-871194

[6] United States, National Institutes of Health, National Cancer Institute (nci). Cancer Trends Progress Report-2009/2010 Update. Bethesda: nci; 2010.

[7] Jemal A, Siegel R, Xu J, Ward E (2010) Cancer statistics. CA Cancer J Clin 60: 277–300. 20610543 10.3322/caac.20073

[8] Thoppil RJ, Bishayee A (2011) Terpenoids as potential chemopreventive and therapeutic agents in liver cancer World J Hepatol. 3: 228–249. 10.4254/wjh.v3.i9.228 PMC3182282 21969877

[9] Bruix J, Sherman M, Llovet JM, Beaugrand M, Lencioni R, et al (2001) Clinical management of hepatocellular carcinoma. Conclusions of the Barcelona-2000 EASL conference. European Association for the Study of the Liver. J Hepatol 35: 421–430. 11592607 10.1016/s0168-8278(01)00130-1

[10] Lu SC (2010) Where are we in the chemoprevention of hepatocellular carcinoma? Hepatology 51: 734–736. 20198627 10.1002/hep.23497

[11] Je Y, Schutz FA, Choueiri TK (2009) Risk of bleeding with vascular endothelial growth factor receptor tyrosine-kinase inhibitors sunitinib and sorafenib: a systematic review and metaanalysis of clinical trials. Lancet Oncol 10: 967–974. 19767240 10.1016/S1470-2045(09)70222-0

[12] Llovet JM, Ricci S, Mazzaferro V, Hilgard P, Gane E, et al (2008) Sorafenib in advanced hepatocellular carcinoma. N Engl J Med 359: 378–390. 18650514 10.1056/NEJMoa0708857

[13] Aggarwal BB, Shishodia S (2006) Molecular targets of dietary agents for prevention and therapy of cancer. Biochem Pharmacol 71: 1397–1421. 16563357 10.1016/j.bcp.2006.02.009

[14] Russo GL (2007) Ins and outs of dietary phytochemicals in cancer chemoprevention. Biochem Pharmacol 74: 533–544. 17382300 10.1016/j.bcp.2007.02.014

[15] Naithani R, Huma LC, Moriarty RM, McCormick DL, Mehta RG (2008) Comprehensive review of cancer chemopreventive agents evaluated in experimental carcinogenesis models and clinical trials. Curr Med Chem 15: 1044–1071. 18473802 10.2174/092986708784221403

[16] Kaefer CM, Milner JA (2008) The role of herbs and spices in cancer prevention. J Nutr Biochem 19: 347–361. 18499033 10.1016/j.jnutbio.2007.11.003 PMC2771684

[17] Moiseeva EP, Manson MM (2009) Dietary chemopreventive phytochemicals: too little or too much? Cancer Prev Res (Phila) 2: 611–616. 19584074 10.1158/1940-6207.CAPR-08-0102

[18] Kris-Etherton PM, Hecker KD, Bonanome A, Coval SM, Binkoski AE, et al (2002) Bioactive compounds in foods: their role in the prevention of cardiovascular disease and cancer. Am J Med 113 Suppl 9B71S–88S. 12566142 10.1016/s0002-9343(01)00995-0

[19] Riboli E, Norat T (2003) Epidemiologic evidence of the protective effect of fruit and vegetables on cancer risk. Am J Clin Nutr 78: 559S–569S. 12936950 10.1093/ajcn/78.3.559S

[20] World Cancer Research Fund/American Institute for Cancer Research. Food, Nutrition, Physical Activity and the Prevention of Cancer: A Global Perspective. Washington, DC: AICR, 2007.

[21] Anand P, Sundaram C, Jhurani S, Kunnumakkara AB, Aggarwal BB (2008) Curcumin and cancer: an “old-age” disease with an “age-old” solution. Cancer Lett 267: 133–164. 18462866 10.1016/j.canlet.2008.03.025

[22] Bae MK, Kim SH, Jeong JW, Lee YM, Kim HS, et al (2006) Curcumin inhibits hypoxia-induced angiogenesis via down-regulation of HIF-1. Oncol Rep 15: 1557–1562. 16685395

[23] Choi H, Chun YS, Kim SW, Kim MS, Park JW (2006) Curcumin inhibits hypoxia-inducible factor-1 by degrading aryl hydrocarbon receptor nuclear translocator: a mechanism of tumor growth inhibition. Mol Pharmacol 70: 1664–1671. 16880289 10.1124/mol.106.025817

[24] Labbozzetta M, Notarbartolo M, Poma P, Giannitrapani L, Cervello M, et al (2006) Significance of autologous interleukin-6 production in the HA22T/VGH cell model of hepatocellular carcinoma. Ann N Y Acad Sci 1089: 268–275. 17261774 10.1196/annals.1386.014

[25] Aggarwal BB, Kumar A, Bharti AC (2003) Anticancer potential of curcumin: preclinical and clinical studies. Anticancer Res 23: 363–398. 12680238

[26] Liang Y, Yin D, Zheng T, Wang J, Meng X, et al (2011) Diphenyl Difluoroketone: A Potent Chemotherapy Candidate for Human Hepatocellular Carcinoma. Plos One 6: e23908. 21901145 10.1371/journal.pone.0023908 PMC3162018

[27] Shoba G, Joy D, Joseph T, Majeed M, Rajendran R, et al (1998) Influence of piperine on the pharmacokinetics of curcumin in animals and human volunteers. Planta Med 64: 353–356. 9619120 10.1055/s-2006-957450

[28] Buhrow SA, Reid JM, Jia L, Mamoru SJ, Snyder JP, et al (2005) LC/MS/MS assay and mouse pharmacokinetics and metabolism of the novel curcumin analog EF-24 (NSC 716993). AACR Meeting Abstracts 2005 2005: 984–a.

[29] Adams BK, Ferstl EM, Davis MC, Herold M, Kurtkaya S, et al (2004) Synthesis and biological evaluation of novel curcumin analogs as anti-cancer and antiangiogenesis agents. Bioorg Med Chem 12: 3871–3883. 15210154 10.1016/j.bmc.2004.05.006

[30] Selvendiran K, Tong L, Vishwanath S, Bratasz A, Trigg NJ, et al (2007) EF24 induces G2/M arrest and apoptosis in cisplatin-resistant human ovarian cancer cells by increasing PTEN expression. J Biol Chem 282: 28609–28618. 17684018 10.1074/jbc.M703796200 PMC4610350

[31] Thomas SL, Zhong D, Zhou W, Malik S, Liotta D, et al (2008) EF24, a novel curcumin analog, disrupts the microtubule cytoskeleton and inhibits HIF-1. Cell Cycle 7: 2409–2417. 18682687 10.4161/cc.6410 PMC2573855

[32] Thomas SL, Zhao J, Li Z, Lou B, Du Y, et al (2010) Activation of the p38 pathway by a novel monoketone curcumin analog, EF24, suggests a potential combination strategy. Biochem Pharmacol 80: 1309–1316. 20615389 10.1016/j.bcp.2010.06.048 PMC3690458

[33] Subramaniam D, May R, Sureban SM, Lee KB, George R, et al (2008) Diphenyl difluoroketone: a curcumin derivative with potent in vivo anticancer activity. Cancer Res 68: 1962–1969. 18339878 10.1158/0008-5472.CAN-07-6011

[34] Adams BK, Cai J, Armstrong J, Herold M, Lu YJ, et al (2005) EF24, a novel synthetic curcumin analog, induces apoptosis in cancer cells via a redox-dependent mechanism. Anticancer Drugs 16: 263–275. 15711178 10.1097/00001813-200503000-00005

[35] Shoji M, Sun A, Kisiel W, Lu YJ, Shim H, et al (2008) Targeting tissue factor-expressing tumor angiogenesis and tumors with EF24 conjugated to factor VIIa. J Drug Target 16: 185–197. 18365880 10.1080/10611860801890093

[36] Zhou Y, Zheng S, Lin J, Zhang QJ, Chen A (2007) The interruption of the PDGF and EGF signaling pathways by curcumin stimulates gene expression of PPARγ in rat activated hepatic stellate cell in vitro. Laboratory Investigation 87, 488–498. 10.1038/labinvest.3700532 17372590

[37] Mayo LD, Dixon JE, Durden DL, Tonks NK, Donner DB (2002) PTEN Protects p53 from Mdm2 and Sensitizes Cancer Cells to Chemotherapy. J Biol Chem. 277: 5484–5489. 10.1074/jbc.M108302200 11729185

[38] Agarwal ML, Agarwal A, Taylor WR, Stark GR (1995) p53 controls both the G2/M and the G1 cell cycle checkpoints and mediates reversible growth arrest in human fibroblasts Proc. Natl Acad Sci U S A. 92: 8493–8497. 10.1073/pnas.92.18.8493 PMC41183 7667317

[39] Deiry WS, Tokino T, Velculescu VE, Levy DB, Parsons R, et al (1993) WAF1, a potential mediator of p53 tumor suppression. Cell 75: 817–825. 8242752 10.1016/0092-8674(93)90500-p

[40] Coqueret O (2003) New roles for p21 and p27 cell-cycle inhibitors: a function for each cell compartment? Trends Cell Biol 13: 65–70. 12559756 10.1016/s0962-8924(02)00043-0

[41] Kluck RM, Bossy WE, Green DR, Newmeyer DD (1997) The release of cytochrome c from mitochondria: a primary site for Bcl-2 regulation of apoptosis. Science 275: 1132–1136. 9027315 10.1126/science.275.5303.1132

[42] Wang X (2001) The expanding role of mitochondria in apoptosis. Genes Dev 15: 2922–2933. 11711427

[43] de Groot RP, Coffer PJ, Koenderman L (1998) Regulation of proliferation, differentiation and survival by the IL-3/IL-5/GM-CSF receptor family. Cell Signal 10: 619–628. 9794243 10.1016/s0898-6568(98)00023-0

[44] Malemud CJ (2007) Inhibitors of stress-activated protein/mitogen-activated protein kinase pathways. Curr Opin Pharmacol 7: 339–343. 17398158 10.1016/j.coph.2006.11.012

[45] Morrison DK, Davis RJ (2003) Regulation of MAP kinase signaling modules by scaffold proteins in mammals. Annu Rev Cell Dev Biol 19: 91–118. 14570565 10.1146/annurev.cellbio.19.111401.091942

[46] Rennefahrt U, Janakiraman M, Ollinger R, Troppmair J (2005) Stress kinase signaling in cancer: fact or fiction? Cancer Lett 217: 1–9. 15596290 10.1016/j.canlet.2004.08.003

[47] Chen A, Xu J, Johnson AC (2006) Curcumin inhibits human colon cancer cell growth by suppressing gene expression of epidermal growth factor receptor through reducing the activity of the transcription factor, Egr-1. Oncogene 25: 278–287. 16170359 10.1038/sj.onc.1209019

[48] Krystal GW, Sulanke G, Litz J (2002) Inhibition of Phosphatidylinositol 3-Kinase-Akt Signaling Blocks Growth, Promotes Apoptosis, and Enhances Sensitivity of Small Cell Lung Cancer Cells to Chemotherapy. Mol Cancer Ther 1: 913–922. 12481412

[49] Liu X, Shi Y, Giranda VL, Luo Y (2006) Inhibition of the phosphatidylinositol 3-kinase/Akt pathway sensitizes MDA-MB468 human breast cancer cells to cerulenin-induced apoptosis. Mol Cancer Ther 5: 494–501. 16546963 10.1158/1535-7163.MCT-05-0049

[50] Liu XH, Kirschenbaum A, Yao S, Lee R, Holland JF, et al (2000) Inhibition of cyclooxygenase-2 suppresses angiogenesis and the growth of prostate cancer in vivo. J Urol 164: 820–825. 10953162 10.1097/00005392-200009010-00056

[51] Kern MA, Hauqq AM, Koch AF, Schilling T, Breuhahn K, et al (2006) Cyclooxygenase-2 Inhibition Induces Apoptosis Signaling via Death Receptors and Mitochondria in Hepatocellular carcinoma. Cancer Res 66: 7059–7066. 16849551 10.1158/0008-5472.CAN-06-0325

[52] Liu F, Wang P, Jiang X, Tan G, Qiao H, et al (2008) Antisense hypoxiainducible factor 1alpha gene therapy enhances the therapeutic efficacy of doxorubicin to combat hepatocellular carcinoma. Cancer Sci 99: 2055–2061. 19016766 10.1111/j.1349-7006.2008.00905.x PMC11159667

[53] Wang J, Ma Y, Jiang H, Zhu H, Liu L, et al (2011) Overexpression of von Hippel–Lindau protein synergizes with doxorubicin to suppress hepatocellular carcinoma in mice. Journal of Hepatology 55: 359–368. 21168458 10.1016/j.jhep.2010.10.043

